# Editorial: New technological frontiers in the diagnosis and treatment of gastrointestinal cancers

**DOI:** 10.3389/fmed.2024.1362631

**Published:** 2024-02-23

**Authors:** Claudio Fiorillo, Giuseppe Quero, Pietro Mascagni, Sergio Alfieri

**Affiliations:** ^1^Agostino Gemelli University Polyclinic (IRCCS), Rome, Italy; ^2^Faculty of Medicine, Università Cattolica del Sacro Cuore di Roma, Rome, Italy; ^3^Institut de Chirurgie Guidée par l'Image (IHU), Strasbourg, France

**Keywords:** gastrointestinal tumor, artificial intelligence, radiomic, fluorescence, precision surgery

## Introduction

In the ever-evolving landscape of medical science, breakthroughs in technology continue to redefine the paradigms of diagnosis and treatment. This compilation of articles, aptly titled “*New technological frontiers in the diagnosis and treatment of gastrointestinal cancers*,” explores the cutting-edge advancements that promise to revolutionize our approach to combating one of the most challenging medical frontiers. From artificial intelligence (AI) in surgery to the utilization of fluorescence and radiomics, these articles collectively illuminate the promising pathways that lie ahead in the battle against gastrointestinal cancers.

## Artificial intelligence: transforming surgical precision

One of the most transformative forces in modern medicine is the integration of artificial intelligence into surgical practices (Zuo et al.). The synergy between human expertise and AI algorithms is enhancing the precision and efficiency of gastrointestinal cancer surgeries. AI applications, such as machine learning algorithms, could assist surgeons in real-time decision-making, enabling more accurate diagnoses and personalized treatment plans.

In the realm of gastrointestinal cancer surgery, AI plays a pivotal role in image analysis, aiding surgeons in identifying subtle anomalies that may elude the naked eye. The ability of AI to analyze vast datasets and recognize patterns contributes to early detection and precise localization of tumors. As a result, patients experience improved outcomes, reduced complications, and shorter recovery times.

## Fluorescence-guided surgery: illuminating the unseen

Fluorescence-guided surgery represents another groundbreaking avenue in the diagnosis and treatment of gastrointestinal cancers. This technique involves the use of fluorescent dyes that selectively bind to cancerous cells, rendering them visible under specific wavelengths of light. This enhanced visibility could empower surgeons to distinguish between healthy and cancerous tissues with unprecedented clarity.

In gastrointestinal cancer surgery, fluorescence-guided techniques enable surgeons to visualize tumor margins in real time. This heightened precision aids in the complete removal of cancerous tissue while minimizing damage of surrounding healthy structures. The potential for improved outcomes and reduced recurrence rates makes fluorescence-guided surgery a promising frontier in the fight against gastrointestinal cancers (Xia et al.).

## Radiomics: harnessing the power of medical imaging

The marriage of radiology and informatics gives rise to radiomics (Miccichè et al.), a field that extracts quantitative data from medical images for comprehensive analysis. In the context of gastrointestinal cancers, radiomics offers a non-invasive approach to characterize tumors based on their imaging features. This wealth of information goes beyond what traditional imaging methods provide, allowing for a more nuanced understanding of tumor biology.

Radiomics holds the promise of transforming the landscape of cancer diagnosis and prognosis. By extracting and analyzing intricate patterns within medical images, radiomics contributes to the development of predictive models that guide treatment decisions. This approach enhances the ability to tailor interventions to individual patient profiles, fostering a more personalized and effective treatment paradigm.

## The interplay of technologies: a holistic approach to gastrointestinal cancer care

While each technological frontier brings its unique advantages, the true power lies in their synergistic application (1). The integration of AI, fluorescence-guided surgery, and radiomics creates a comprehensive approach to gastrointestinal cancer care (Zeng et al.). AI-driven analyses of radiomic data can inform surgeons during fluorescence-guided procedures, ensuring a meticulous and personalized surgical intervention.

Furthermore, the data generated from these technologies contribute to a growing pool of knowledge that fuels ongoing research and innovation (Khalid et al.). The insights gained from the interplay of these technologies not only refine current practices but also lay the groundwork for the next generation of advancements in gastrointestinal cancer diagnosis and treatment.

## Challenges and ethical considerations

As we celebrate the strides made in technological frontiers, it is essential to acknowledge the challenges and ethical considerations that accompany these innovations. Ensuring the responsible and ethical use of AI in surgery, addressing potential biases in algorithms, and safeguarding patient privacy are paramount. Moreover, the introduction of novel technologies necessitates ongoing collaboration between medical professionals, researchers, and ethicists to establish guidelines that prioritize patient wellbeing.

## Conclusion

The articles presented in this compilation collectively underscore the transformative potential of AI, fluorescence-guided surgery, and radiomics in the diagnosis and treatment of gastrointestinal cancers. As we stand on the brink of a new era in medical science, these technological frontiers promise to reshape our approach to cancer care, offering hope to patients and practitioners alike. Through continued research, collaboration, and ethical considerations, we can navigate these frontiers responsibly, ensuring that the future of gastrointestinal cancer care is characterized by precision, compassion, and improved outcomes for all.

## Author contributions

CF: Conceptualization, Project administration, Writing – original draft, Writing – review & editing. GQ: Conceptualization, Writing – original draft, Writing – review & editing. PM: Methodology, Writing – original draft, Writing – review & editing. SA: Supervision, Writing – original draft, Writing – review & editing.
